# Compression of the Inferior Alveolar Canal by Mandibular Third Molar among Images Taken from Patients Visiting Dental Imaging Centres of Kathmandu: A Descriptive Cross-sectional Study

**DOI:** 10.31729/jnma.7124

**Published:** 2022-01-31

**Authors:** Rinky Nyachhyon, Ujjwal Joshi, Apeksha Mainali, Pranay Sakya

**Affiliations:** 1 Department of Oral Medicine and Radiology, People's Dental College and Hospital, Sorhakhutte, Kathmandu, Nepal; 2 Department of Oral Medicine and Radiology, Kathmandu Medical College, Duwakot, Bhaktpur, Nepal; 3 Department of Oral Medicine and Radiology, Nepal Medical College, Attarkhel, Kathmandu, Nepal; 4 Department of Oral Surgery, People's Dental College and Hospital, Sorhakhutte, Kathmandu, Nepal

**Keywords:** *cone beam computed tomography*, *inferior alveolar nerve*, *mandible*, *third molar*

## Abstract

**Introduction:**

Third molars are common teeth to be impacted. The position of mandibular third molar is such that it is in close contact with inferior alveolar canal which may lead to nerve damage during its removal. So, this study was conducted to find out the prevalence of compression of inferior alveolar canal by mandibular third molars.

**Methods:**

This descriptive cross-sectional study was conducted on images collected from Dental Imaging Centers of Nepal from 25^th^ June 2020 to 15^th^ February 2021 after obtaining ethical clearance from Nepal Health Research Council (Reference number: 2100). A convenience sampling method was used to collect 433 cone-beam computed tomography images showing the relation between the third molars and inferior alveolar canal. Data were analyzed using the Statistical Package for the Social Sciences version 16. Point estimate at 95% confidence interval was calculated along with frequency and proportion for the binary data.

**Results:**

Out of 433 images, 135 (31.17%) (26.80-35.53 at 95% Confidence Interval) images showed compression of inferior alveolar nerve by mandibular third molar. The study result indicated that 16 (11.85%) buccally placed, 50 (37.03%) lingually placed and 69 (51.11%) inferiorly placed inferior alveolar canal were compressed by apices of mandibular third molars.

**Conclusions:**

The prevalence of compression of inferior alveolar canal by mandibular third molar was found to be similar to other studies done in similar settings. Compression of the canal was more evident when inferior alveolar canal is situated lingually.

## INTRODUCTION

Mandibular third molars (MM3s) are last teeth to erupt in oral cavity, 40% of which fail to erupt, mainly due to lack of space.^1^ They are associated with various complications like pericoronitis, odontogenic cyst, dental crowding.^1,2^ MM3 roots can contact or penetrate into mandibular canal where inferior alveolar nerve is enclosed. This close relationship can damage inferior alveolar nerve during surgical procedures.^3-6^ Damage is common when roots of MM3 lie in buccal and inferior position.^7^

Orthopantomogram (OPG) is common diagnostic radiograph taken before MM3 surgery.^8-12^ OPG being two-dimensional cannot accurately determine number of roots, morphology, exact location of inferior alveolar nerve.^6,13,14^ However, cone beam computed tomography (CBCT) provides three-dimensional volumetric data reconstruction and can illustrate buccolingual position of MM3 in relation to inferior alveolar canal without any distortion.^8,15,16^

Hence, the objective of this study was to find the prevalence of compression of inferior alveolar canal by mandibular third molars on CBCT.

## METHODS

This descriptive cross-sectional study was carried out after receiving the approval from Nepal Health Research Council (Reference no. 2100). The data collection was done from 25^th^ June 2020 to 15^th^ February 2021. CBCT images were collected from patients who visited Dental Imaging Centre (DIC), Metro Radiology and Imaging P. Ltd (MRI), and People's Dental College and Hospital (PDCH) for their regular diagnosis and treatment planning. Images included in study showed clearly the relation between the third molars and inferior alveolar canal. Images excluded from the study were MM3 with periapical pathology and third molars with incomplete root formation.

Sample size was calculated using the formula,

n = Z^2^ × (p × q) / e^2^

  = (1.96) ^2^ × 0.5 × (1-0.5) / (0.05)^2^

  = 385

Where,

n= required sample size
Z= 1.96 at 95% Confidence Interval (CI)
p= prevalence of compression of inferior alveolar canal by mandibular third molars taken as 50% for maximum sample size
e= margin of error, 5% in this study

The minimum required sample size was 385, but 433 images of MM3 region were included for the study after taking written informed consent.

The images were analysed in Planmeca Romexis and Galaxis 3D software. Serial sagittal, coronal, and axial images were studied independently. The images were evaluated carefully by continuously moving the toolbar until the proper relation of MM3 with inferior alveolar canal was evident. The compression of the inferior alveolar canal by mandibular third molars was assessed. Also recorded were the observations whether the mandibular canal was buccal, lingual, or inferior to the roots of third molars.

Data were analysed using the Statistical Package for the Social Sciences version 16. Point estimate at 95% confidence interval was calculated along with frequency and proportion for the binary data.

## RESULTS

**Figure 1:**
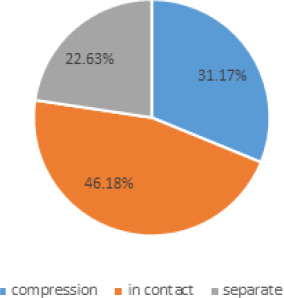
Relation of inferior alveolar canal with mandibular third molar

Out of 433 images the inferior alveolar canal was compressed by MM3 in 135 (31.17%) (26.80-35.53 at 95% Confidence Interval) of cases. In 200 (46.18%) inferior alveolar canal were in contact and in 98 (22.63%), inferior alveolar canal was separate from MM3 (Figure 1).

The study result indicated that 16 (11.85%) buccally placed, 50 (37.03%) lingually placed and 69 (1.11%) inferiorly placed inferior alveolar canal were compressed by apices of mandibular third molars. In 200 (46.18%) images, the inferior alveolar canal was just in contact with the MM3, whereas the inferior alveolar canal was not in contact with MM3 in 98 (22.63%) images. About 233 (53.8%) images revealed inferior alveolar canal to be in the inferior position. Buccal position of the canal was the second most common and lingual position was the least common observed in only 78 (18%) patients.

The number of lingually placed canals were very low, however the canal compression was recorded in 50 (37.03%) when the canal assumed lingual position relative to MM3. In buccal position, the mandibular canal was less likely to cause compression, seen in 16 (11.85%) cases (Table 1).

**Table 1:** Relation of third molars to inferior alveolar canal according to side (n=135).

Side	Compressed n (%)
Buccal	16 (11.85)
Lingual	50 (37.03)
Inferior	69 (51.11)

There were almost equal number of male and female in our study that is 220 (50.8%) males and 230 (49.2%) female. Compression of inferior alveolar canal with MM3 was also evaluated among male and female. Among images of the male 59 (26.8%) cases showed compression of the nerve by the root of MM3 whereas in 76 (35.7%) cases images of the female showed compression. Age was further classified into groups to see the compression of nerve according to age (Table 2).

**Table 2:** Relation of third molars to inferior alveolar canal according to age (n=135).

Age (in years)	Compressed n (%)
< 20	16 (11.85)
20-39	102 (75.55)
40-59	16 (11.85)
≥60	1 (0.74)

## DISCUSSION

Inferior alveolar nerve damage can be due to various causes like implant, orthognathic surgery and third molar removal. The main cause of inferior alveolar nerve damage is because of removal of impacted third molar.^10^

A preoperative radiograph is mandatory to know the exact relation of the third molars to the inferior alveolar canal to avoid the post-operative damage to inferior alveolar nerve.^2,11^ OPG are the most common preoperative radiograph taken prior to the removal of third molars. Several studies have indicated that the seven radiographic findings may indicate the proximity of inferior alveolar canal with the roots: darkening of root apex, deflection of root, narrowing of root, presence of bifid root, interruption of white line of inferior alveolar canal, diversion of inferior alveolar canal, and narrowing of inferior canal.^3,6,9,17^ However, the presence of these radiographic signs doesn't confirm the exact location of the roots with the canal^7,15^ and the possibility of injury to inferior alveolar nerve.^2^ The absence of these signs do not always indicate that there is no direct contact of mandibular third with mandibular canal.^10^

Panoramic radiograph failed to identify the exposure of inferior alveolar nerve exposure in nearly one third of cases.^13^ However, cone beam computed tomography (CBCT) provides three-dimensional volumetric data reconstruction and can illustrate buccolingual position of the tooth in relation to inferior alveolar canal without any distortion^8^ with low exposure doses compared to CT.^2,8,13^ CBCT are reliable and accurate for clinical measurements.^13,15^ Also, Tomasi, et al^18^. and Nikneshan, et al^19^. proved in their study that linear measurements of CBCT is highly reliable.

Ghaeminia, et al. found no significant differences in sensitivity and specificity between the CBCT and OPG in predicting inferior alveolar nerve exposure.^11^ However other studies proved CBCT to be better radiographic modality compared to OPG.^2,4,9,13^ After many studies it has been concluded that if in OPG, the MM3 appears close to inferior alveolar canal, CBCT is recommended, however, if the third molars don't appear close to inferior alveolar canal, OPG is sufficient to plan for removal of third molars.^2,6,8,12^ In our context, patient are advised CBCT prior to third molar extraction only if the third molars appear close or encroaching the inferior alveolar canal. So, all the CBCT images of our study were taken only when a third molar appeared close to the inferior alveolar canal in 2D radiographs.

In our study images evaluated included of 49.2% female and 50.8% of males in contrast to studies of Nayak, et al.^4^, Karnasuta, et al.^6^, Kutsson, et al., ^20^ Kovisto, et al.^21^ where images of females were higher in number. The mean age of our study was 29.7 years which is comparable to Kutsson, et al.^20^ where the mean age was 28 years. This study included almost equal number of right and left MM3s which was consistent with result of previous study of Jadu, et al.^7^

On evaluating the position of the inferior alveolar canal with respect to the roots of MM3, our study showed that most canals were located inferior to the inferior alveolar canal which in similar to the studies by Gu, et al.^22^ and Chaudhary, et al.^23^ but in contrast to study by Ghaeminia, et al.^11^ and Karnasuta, et al.^6^ where in most cases, the inferior alveolar canals had lingual position. Less number of cases with lingual position of canals were seen in studies by Jadu, et al.^7^, Gu, et al.^22^, Chaudhary, et al.^23^ and Maglione, et al.^24^ Compression of inferior alveolar canal by the roots of MM3 was maximum 64.1% when the inferior alveolar canal was in lingual position in our study. Chaudhary, et al.^23^ reported the maximum number compression when inferior alveolar canal was placed lingually and Jadu, et al.^7^ reported the maximum compression when the canal was inferior to the MM3.

Since our study is limited to imaging centres of Kathmandu valley only, the findings cannot be generalized. Also, the descriptive nature of this study couldn't determine associations and causality. Further, a larger sample size and random sampling technique could improve the generalizability of this study.

## CONCLUSIONS

The prevalence of compression of inferior alveolar canal by mandibular third molar was found to be similar to other studies done in similar settings. Compression of the canal was more evident when inferior alveolar canal is situated lingually. Cone Beam Computed Tomography provides vital relation between mandibular third molar and the inferior alveolar nerve in all three directions which assists in estimating risk and can also be beneficial for communicating with the patients regarding the best treatment plan for him/her.
